# Intense autumnal coastal biogenic particle settling fluxes align with phytoplankton phenology changes off the western Antarctic Peninsula

**DOI:** 10.1038/s41598-025-92914-9

**Published:** 2025-03-23

**Authors:** Enrique Isla, Eduardo Menschel, Humberto H. González

**Affiliations:** 1 Instituto de Ciencias del Mar-CSIC, Barcelona, Spain; 2 Corporación Regional de Investigación y Desarrollo Cooperativo, Centro de Investigación en Ecosistemas de la Patagonia, CIEP, Coyhaique, Chile; 3 Centro de Investigación Dinámica de Ecosistemas Marinos de Altas Latitudes (IDEAL), Instituto de Ciencias Marinas y Limnológicas, Universidad Austral de Chile, Valdivia, Punta Arenas, Chile

**Keywords:** Antarctic Peninsula, Marine biogeochemical cycles, Marine particle fluxes, Climate change, Organic carbon, Biogenic silica, Ecology, Biogeochemistry, Ecology, Environmental sciences

## Abstract

Autumnal settling particle fluxes were studied in the Antarctic coastal zone. The study revealed that the particulate organic carbon (POC) flux exported from the euphotic zone towards the seabed equals the magnitude of the summer pulse when typically, > 95% of the Antarctic annual flux develops. The pelagic POC flux was accompanied with centric and pennate diatoms and euphausiid faecal pellets, which together comprised most of the biogenic particles (and biogenic silica) collected in the sediment trap used in the Gerlache Strait. Our results strongly suggest that the most important drivers of the unusual seasonal extension observed for the settling particle export were increasing glacier melting and an extended productive period. The present study may provide a baseline for Antarctic coastal biogenic particle flux studies and shows that ongoing environmental warming makes the autumnal biogenic settling particle production near shore more intense than typically observed off shore.

**Supplementary Information:**

The online version contains supplementary material available at 10.1038/s41598-025-92914-9.

## Introduction

Global change is rapidly modifying the marine environment in the vicinity of the Antarctic Peninsula (AP), producing effects that likely will occur in the future elsewhere in the Antarctic^1^. Some of these anthropogenic effects, such as altered biogeochemical cycles, may have already changed the AP ecosystem before there is sufficient scientific evidence (e.g., lack of long-term series to contrast changes) to explain how the unaltered ecosystem used to be. This knowledge gap developed because the time required to study Antarctic coastal ecosystem processes couldn’t cope with the ongoing anthropogenic environmental change pace. The fluxes of settling particulate mass (e.g., biogenic and lithogenic matter, including the organic carbon that feeds marine benthos), developing at the Antarctic coastal environment may be one example for it. Estimating the magnitude of marine biogenic particle settling fluxes (BPSF) provides fundamental information on the transport of mass (e.g., different microplankton functional groups, zooplankton faecal pellets –zFP-, phytodetritus aggregates) and energy along the marine food web and biogeochemical cycle dynamics. Studying BPSF is also important to accurately calculate the global carbon budget and assess atmospheric carbon sequestration, which has important implications for global climate regulation. Polar climate makes marine Antarctic environmental conditions markedly seasonal, with illuminated open water and high primary production (PP) rates during the late spring and summer in contrast to dense sea-ice coverage under weak light and negligible PP during the autumn and winter. This contrast is also observed in the annual pattern of settling mass and particulate organic carbon (POC) fluxes, including biogenic particulate matter such as silicified diatom aggregates, zFP and planktonic carbonated shells^2–4^. Thus, more than 95% of the total mass (seston), POC and biogenic silica (bSi) export fluxes from the Antarctic euphotic zone towards the seabed occurs during the spring and summer (considering the astronomical equinox and solstice dates), unless the extraordinary presence of icebergs or massive zooplankton schools (e.g., pteropods) modify this typical annual pattern^5–8^. The western Antarctic Peninsula (WAP) is one of the regions on Earth, where global warming increases at relatively faster pace^9^ with cascading effects on marine physical and biological processes, which are not fully understood yet^1,10^. Changes in Antarctic marine pelagic biota have been observed in the last decades with concomitant impacts on the biogeochemical cycles of carbon and silicon^1,11–13^. For example, temperature increases in the region, on the one hand, reduce the sea-ice cover season, increasing the potential for PP to develop^14,15^, whereas on the other, modify the spatial distribution of plankton^16–19^ and consequently, the BPSF from the euphotic zone towards the sea floor. These fluxes are mainly carried down by marine snow, phytoplankton aggregates and zFP (e.g., salps and krill)^3,4,20,21^. Hence, the chemical characteristics of the BPSF are tightly linked to the composition of the planktonic community assemblage. In areas with diatom-dominated communities, the flux of bSi will be higher relative to those areas where non-siliceous species (e.g., *Phaeocystis antarctica*) are more abundant (e.g., Bransfield Strait vs. NW Weddell Sea)^22–24^. In addition, the POC export pattern from the euphotic zone depends on whether krill or salps dominate the zooplankton community abundance, because euphausiid faecal pellets (eFP) are a key contributor to the POC settling flux in the WAP and altering its current majority may modify the POC (e.g., energy) transport system to the seabed and along the marine trophic web^23,25,26^. POC and bSi sinking particle fluxes are commonly linked because diatoms, usually the main constituent of the phytoplankton community, thrive by producing siliceous frustules^27–29^. However, differential preservation conditions in the Antarctic environment decouple bSi and POC abundances, where the concentration of the former increases relative to the latter, as particles move from the euphotic zone into the sea floor sediment^30–33^, making the Antarctic continental margin sediment the deposit of ~ 3% of the global ocean’s sedimentary bSi^28^.

**Fig. 1:**
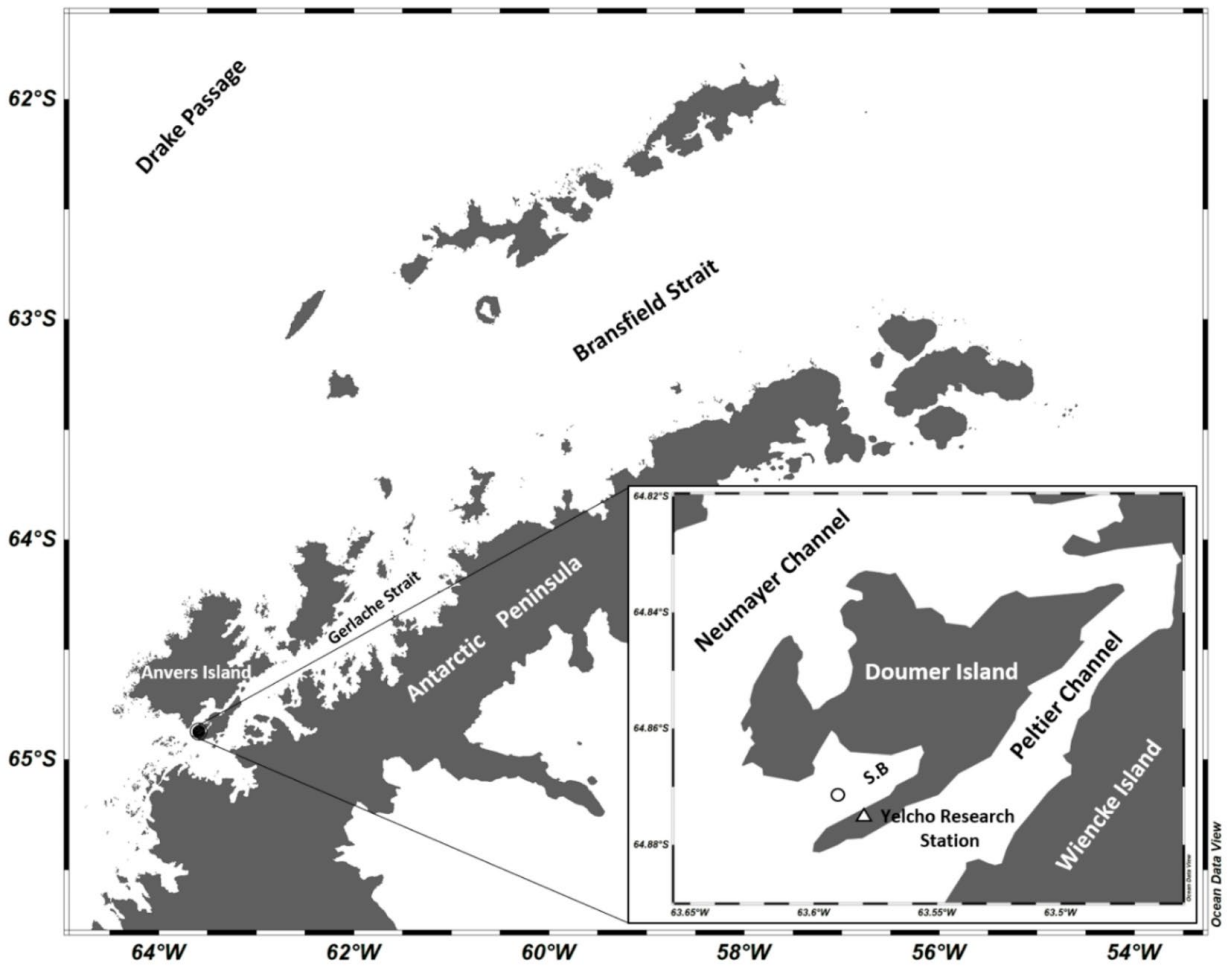
Study area showing the Doumer Island’s South Bay (SB) off the western Antarctic Peninsula. The circle marks the mooring site.

In contrast to offshore observations, little is known about BPSF in the Antarctic coastal zone, specially beyond the spring–summer phytoplankton bloom season^13,34^, mainly because the transit of icebergs strongly limits the installation of moored instruments to acquire long-term information (e.g., years) on environmental variables (e.g., particle fluxes) in near-shore Antarctic settings. The rugged coast line of the WAP enables long water residence times and water column stability, two factors that enhance PP near the shore^33^. This is especially important because wind stress forces lateral transport of particulate organic matter offshore, eventually making the highly productive coastal zone a mass and energy exporter to the adjacent oceanic areas^35^, contributing to maintain biological production levels and atmospheric carbon drawdown offshore^33^. The present study shows results of BPSF acquired during the autumn (beyond the equinox) in an Antarctic coastal setting, the Doumer Island’s South Bay (DISB) in the Gerlache Strait (Fig. 1). This investigation provides the opportunity to get insights into the seasonal state of a system at the onset of a drastic reduction in its biologically productive pace, after the vigorous spring–summer production period^36^. The study attempted to analyze the annual variation in BPSF in the coastal Antarctic; however, due to instrumental failure, the results were constrained to the summer-autumn period providing nevertheless, high-resolution (7–45 days) observations, for an Antarctic near-shore environment. The results showed that the magnitude of Antarctic autumnal BPSF is similar to the observed values for the late summer, implying that the autumnal biological production represents a continuation of the estival pace. This relatively longer (beyond summer) biologically productive time span is still an unconsidered characteristic of the mass and energy budgets for the coastal Antarctic. The present results contribute to accurately estimate temporal variations in BPSF in the Antarctic coastal pelagic system and the marine POC and bSi budgets, in combination with changes in the planktonic community abundances and zFP production. The findings also set the spotlight over the urgent need for the establishment of long-term (e.g., decadal) studies in a region where climate change may have already affected the ecosystem afore knowing which was the situation before the current warming trend and its effects (e.g., diminishing sea ice extent and persistence, increasing glacier runoffs) set in^14^. Based on the observed coastal settling particulate bSi and OC autumnal fluxes, the question arises on whether the observed autumnal BPSF for the DISB represent already an effect of the current ocean warming, and consequently, an extent in time of the pelagic summertime phytoplankton bloom (e.g., alteration of the coastal AP phenology) and its cascading effects on the marine trophic web^13^.

## Results and discussion

### Mass and energy fluxes in the near-shore Antarctic

**Fig. 2:**
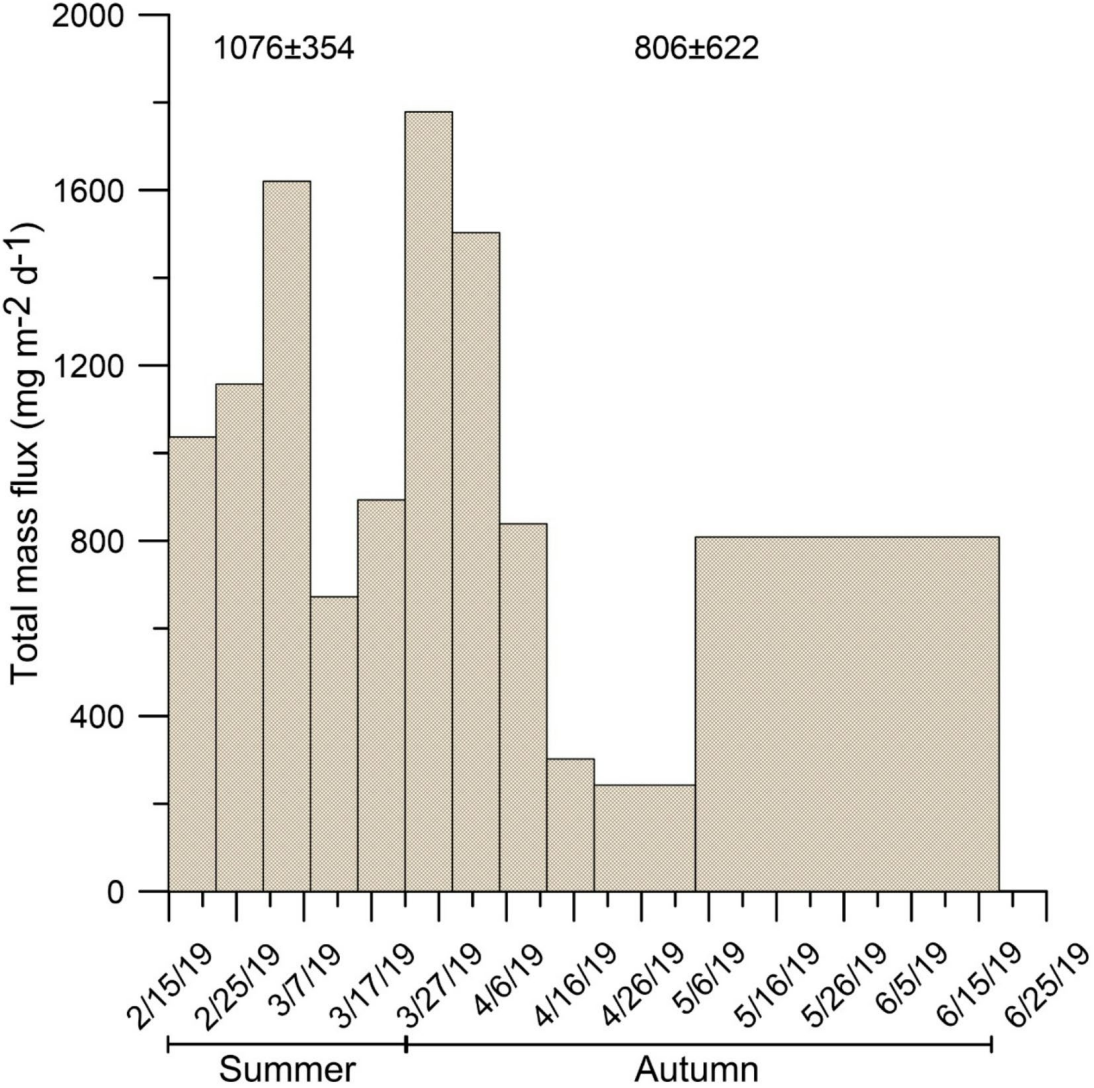
Total mass flux collected in DISB. The numbers above the histogram indicate the seasonal averages.

**Fig. 3:**
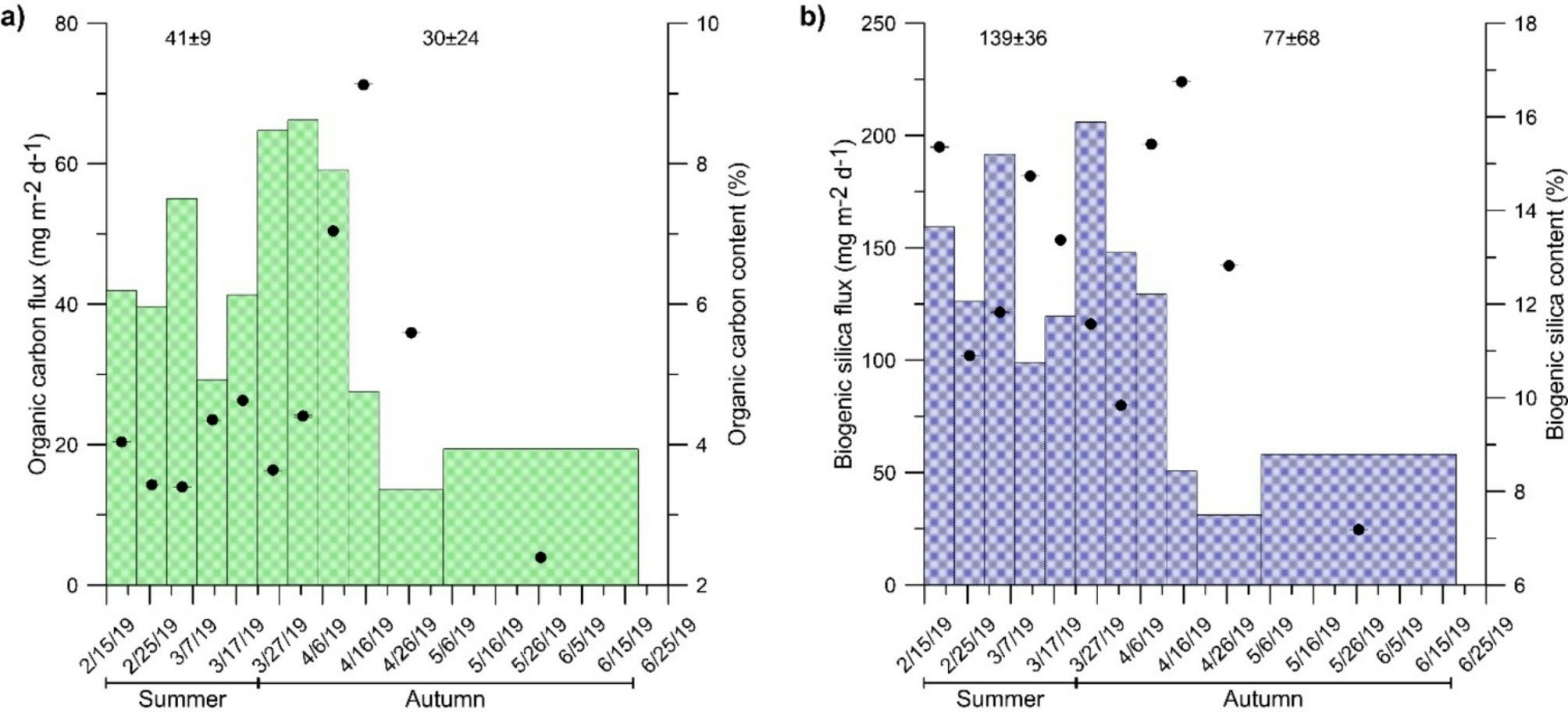
Particulate organic carbon (**a**) and biogenic silica (**b**) fluxes collected in DISB. The numbers above the histogram indicate the seasonal averages. The right X axis and the black dots show the relative contribution of each variable to the correspondent flux.

In DISB, the total mass flux (TMF) varied from 243 to 1778 mg m^−2^ d^−1^ (Fig. 2), whereas the ranges for POC and bSi fluxes, were 14 mg m^−2^ d^−1^ and 66 mg m^−2^ d^−1^ (Fig. 3a) and 31 mg m^−2^ d^−1^ and 206 mg m^−2^ d^−1^, respectively (Fig. 3b). The seasonal averages of total mass, POC (e.g., energy that feed marine benthos) and bSi settling fluxes showed not statistically significant differences among summer and autumn, considering their astronomical temporal limits marked by the solstice and the equinox, respectively (Figs. 2 and 3, Supplementary Table 1). These results suggested that, at least to the south of the Gerlache Strait (central WAP), summer biogenic settling particle production in the coastal Antarctic extents into the autumn and consequently, BPSF during this season are potentially as important as the summer fluxes in providing biogenic matter and energy to both near-shore and offshore areas. Further, the observed autumnal fluxes for the DISB (e.g., 806 mg m^−2^ d^−1^, 30 mg m^−2^ d^−1^, 77 mg m^−2^ d^−1^ for TMF, POC and bSi, respectively) were higher (in some cases as high as an order of magnitude) than most of the observed values for the Antarctic intermediate water depth (> 400 m) autumnal offshore environment (0.5 to 234 mg m^−2^ d^−1^, 0.4 to 21 mg m^−2^ d^−1^, 0.1 to 137 mg m^−2^ d^−1^ for TMF, POC and bSi, respectively), where seabed resuspended sediment into the sediment trap collections has no influence (Figs. 2 and 3, Tables 1 and 2). In contrast, in Antarctic near-shore and near-seabed environments off the Deception and Livingston Islands in the Bransfield Strait (for Livingston Island only summer values available), the observed TMF, POC and bSi settling flux values were one order of magnitude larger than those collected in DISB (3444 to 42,857 mg m^−2^ d^−1^, 20 to 206 mg m^−2^ d^−1^ and 962 mg m^−2^ d^−1^, for TMF, POC and bSi, respectively). The difference is attributed to seabed sediment resuspension, which could increase the TMF magnitudes, collected 4.5 m above the seabed (mas) in Livingston Island^37^ and 20 mas in Deception Island^34^. Particularly, in the Deception Island study^34^, a second sediment trap on the same mooring line was tethered at 50 mas to avoid sediment resuspension effects on TMF collections. Taking this and other precautions^38,39^ in the present study, the sediment trap was also set at 50 mas. The shallower sediment trap at Deception Island collected higher TMF than those observed for DISB, specially during July and August (winter fluxes), when particle fluxes were attributed to ice-rafted debris and wind-borne sources^34^. However, the autumnal fluxes at 50 mas in Deception Island were similar to those observed for DISB, corroborating that the effect of seabed resuspended sediment into the DISB sediment trap collections was negligible. In DISB, POC and bSi mass weights, varied between 2.4 and 9.1% and 7.2 and 16.8% of the TMF, respectively (Fig. 3a and b). These percentages were lower than the values found in open-water, intermediate-depth environments (e.g., 179 m water depth on a 407 m water depth study site), where sediment resuspension was negligible and the proportions varied between 9 and 33% for OC and 11 to 48% for bSi^40^. This comparison suggested that a lithogenic particle input diluted the biogenic fraction concentrations of the pelagic sources in DISB sediment trap collections. Further support for this idea comes from the DISB TMF average values, which were an order of magnitude larger than the summer TMF collected elsewhere at deep and intermediate-depth Antarctic oceanic waters (> 400 m water depth and 400 > mas), where insular and continental lithogenic inputs are negligible (Table 1)^2–4,40^. Accordingly, a lithogenic sediment input different to seabed sediment resuspension, should have increased the DISB TMF values. The DISB POC and bSi proportions were higher than the values of the material collected near shore and near the seabed (4.5 mas) in Livingston Island (e.g., 0.3 to 0.8% for POC and 1.8% and 3.1% for bSi)^37^. In that study, the TMF difference among open-water and near-shore environments was attributed to the diluting effect of resuspended sediment (near-seabed effect) and the lithogenic matter derived from glacier melting (near-shore effect) on the biogenic matter concentrations^37^. In the absence of sediment resuspension, the difference in TMF magnitudes among Livingston Island and DISB studies, suggest that the sediment trap collections in DISB were receiving lithogenic particles derived from glacier runoffs. For example, glacier-derived sediment inputs into near-shore sediment trap collections has been observed for the Adelaide Island to the south of the WAP^36^. The absence of a clear TMF trend (or statistically significant differences among summer and autumn) in DISB, indicated that lithogenic matter inputs into the bay and the sediment trap were constant at least until the end of the autumn (winter solstice). Glacier runoff appear as a feasible lithogenic sediment source because this process provides sediment to the adjacent marine water column and even fertilizes it with micronutrients such as iron^41^, developing hotspots of biological production^35,42,43^. Glacier-derived sediment act as ballast accelerating the POC sinking rates and reducing time for remineralization of organic matter, which eventually reduces the POC flux towards the seabed^44,45^. Consequently, sediment trap collections in comparatively shallow environments, should show higher mass and POC fluxes relative to those collected in deeper waters (Table 1), contributing to make the magnitude of coastal settling particle fluxes higher than in off-shore deeper areas^36^. Glacier melting was already observed for DISB during the summer of 2017^35^ and, based on the present TMF results, presumably the same process took place in the summer of 2019 and continued into the autumn. The comparison among δ^13^C and δ^15^N signals in Southern Ocean’s suspended and seabed surface sediments and DISB sediment trap samples, showed that DISB samples (ca. − 28 ± 1.1 δ^13^C‰ and − 10 ± 0.7 δ^15^N‰) matched better with δ^13^C and δ^15^N signals of suspended sediment (ca. − 28 δ^13^C‰ and − 4 δ^15^N‰) than with surface sediment (ca. − 23 δ^13^C‰ and 5 δ^15^N‰) (Supplementary Fig. 1)^46,47^, providing further evidence to demonstrate the input of glacier-derived sediment into the bay. The settling POC flux in DISB was higher than elsewhere in the Antarctic autumn, only comparable to the values found in the other near-shore shallow environment study off Deception Island^34^ (Table 1, Fig. 3a), whereas the DISB bSi flux, was higher than in any other shallow setting (< 200 m water depth) and similar to deep-water stations in the Ross and Weddell Seas (Table 1, Fig. 3b). These comparisons show the potential of WAP coastal areas as mass and POC (e.g., energy) providers for the open sea and also indicate that currently, the autumn in WAP coastal areas may not be as dormant as it is in the Antarctic open water regarding settling biogenic particle production. Further, in DISB, the δ^13^C signal, the POC to N molar ratio and bSi to POC weight ratio during the complete study period were similar to typical phytoplankton values of ca. -28 δ^13^C‰, 6 and 2, respectively (Supplementary Figs. 1 and 2), indicating that most of the biogenic matter collected by the trap had planktonic origin^25,46,48^. Particularly, the centric diatom flux was significantly correlated to the δ^13^C signal (Supplementary Fig. 1 and Supplementary Table 1).

**Table 1:** Autumnal organic carbon fluxes into sediment traps tethered at < 800 m water depth and > 50 m above the seabed in Antarctic settings.

Location/sediment trap water depth (m)	Season	Total mass flux average (mg m^−2^ d^−1^)	Organic carbon flux average (mg m^−2^ d^−1^)	Organic carbon content (%)	Maximum organic carbon content (%)	References
South Bay, Doumer Island/190	Autumn	806 ± 622	30 ± 24	4 ± 2	9	Present study
Deception Island/110	Autumn	3444 ± 4320	20 ± 17	1 ± 0.2	1	Baldwin and Smith^33^
Bransfield Strait/500	Autumn	0.5 ± 0.5	0.042 ± 0.03	11 ± 4	15	Palanques et al.^4^^₸^
King George Island/494	Autumn	6 ± 5	2 ± 2	na	na	Wefer et al.^49^^╬^
WAP/170	Autumn	21 ± 11	9 ± 8	na	na	Ducklow et al.^50^
WAP/170	Autumn	na	4 ± 8	na	na	Ducklow and Stammerjohn^51^
Pridz Bay/460	Autumn	na	4 ± 1	na	5	Yang et al.^52^^╬^
Amundsen Sea/400	Autumn	125 ± 170	8 ± 11	7 ± 1	8	Kim et al.^53^ ^ɶ^
Amundsen Sea/410	Autumn	30 ± 23	2 ± 1	12 ± 7	18	Kim et al.^53^ ^ɶ^
Amundsen Sea/490	Autumn	21 ± 12	2 ± 1	11 ± 6	19	Kim et al.^53^ ^ɶ^
Amundsen Sea/350	Autumn	23 ± 14	4 ± 3	na	na	Ducklow et al.^50^
Ross Sea/230 (B)	Autumn	108 ± 102	14 ± 12	14 ± 1	15	Dunbar et al.^3^
Ross Sea/519 (B)	Autumn	48 ± 100	6 ± 13	14 ± 2	17	Dunbar et al.^3^
Ross Sea/719 (A)	Autumn	190 ± 260	7 ± 11	4 ± 1	6	Dunbar et al.^3^
Ross Sea/360 (2005)	Autumn	232 ± 238	12 ± 11	3 ± 1	5	Chiarini et al.^8^^╬^
Ross Sea/360 (2008)	Autumn	234 ± 144	21 ± 11	8 ± 3	12	Chiarini et al.^8^^╬^
Ross Sea/546	Autumn	26 ± 3	2 ± 1	6 ± 1	6	Langone et al.^54^^╬^
Weddell Sea/360	Autumn	85 ± 75	4 ± 3	na	na	Wefer and Fischer^55^^╬^

WAP stands for western Antarctic Peninsula. *bottom trap, nd = not determined, na = not available, ^╬^values deducted from bar chart, ^₸^reported value, ^ɶ^average estimated from table.

**Table 2:** Autumnal biogenic silica fluxes into sediment traps tethered at < 800 m water depth and > 50 m above the seabed in Antarctic settings.

Location/water depth (m)	Season	Total mass flux average (mg m^−2^ d^−1^)	Biogenic silica flux average (mg m^−2^ d^−1^)	Biogenic silica content (%)	Maximum biogenic silica content (%)	References
South Bay, Doumer Island/190	Autumn	806 ± 622	77 ± 68	10.1 ± 3.5	17	Present study
Bransfield Strait/500	Autumn	0.4 ± 0.5	0.1 ± 0.07	26. 3 ± 9.7	36	Palanques et al.^4^^₸^
Amundsen Sea/400	Autumn	20 ± 15	12 ± 8	48 ± 12	60	Kim et al.^56^ ^ɶ^
Amundsen Sea/400	Autumn	125 ± 170	81 ± 122	58 ± 12	70	Kim et al.^53^ ^ɶ^
Amundsen Sea/410	Autumn	30 ± 23	8 ± 3	21 ± 6	28	Kim et al.^53^ ^ɶ^
Amundsen Sea/490	Autumn	21 ± 12	3 ± 1	13 ± 3	15	Kim et al.^53^ ^ɶ^
Ross Sea/231	Autumn	108 ± 102	44 ± 51	33 ± 20	48	Dunbar et al.^3^^╬^
Ross Sea/519	Autumn	48 ± 100	18 ± 36	32 ± 7	40	Dunbar et al.^3^^╬^
Ross Sea/719	Autumn	190 ± 260	137 ± 190	71 ± 12	86	Dunbar et al.^3^^╬^
Ross Sea/360 & 770* (2005)	Autumn	232 ± 238 337 ± 263*	78 ± 118 136 ± 139*	23 ± 20 29 ± 19*	50 51	Chiarini et al.^8^^╬^
Ross Sea/360 & 770* (2008)	Autumn	234 ± 144 142 ± 12*	66 ± 60 35 ± 11*	26 ± 11 25 ± 7*	35 36	Chiarini et al.^8^^╬^
Ross Sea/546	Autumn	26 ± 3	11 ± 3	41 ± 6	45	Langone et al.^54^^╬^
Weddell Sea/360	Autumn	85 ± 75	67 ± 65	na	na	Wefer and Fischer^55^^╬^

WAP stands for western Antarctic Peninsula. *bottom trap, nd = not determined, na = not available, ^╬^values deducted from bar chart, ^₸^reported value, ^ɶ^average estimated from table.

### Planktonic community composition and pelagic-benthic coupling

**Fig. 4:**
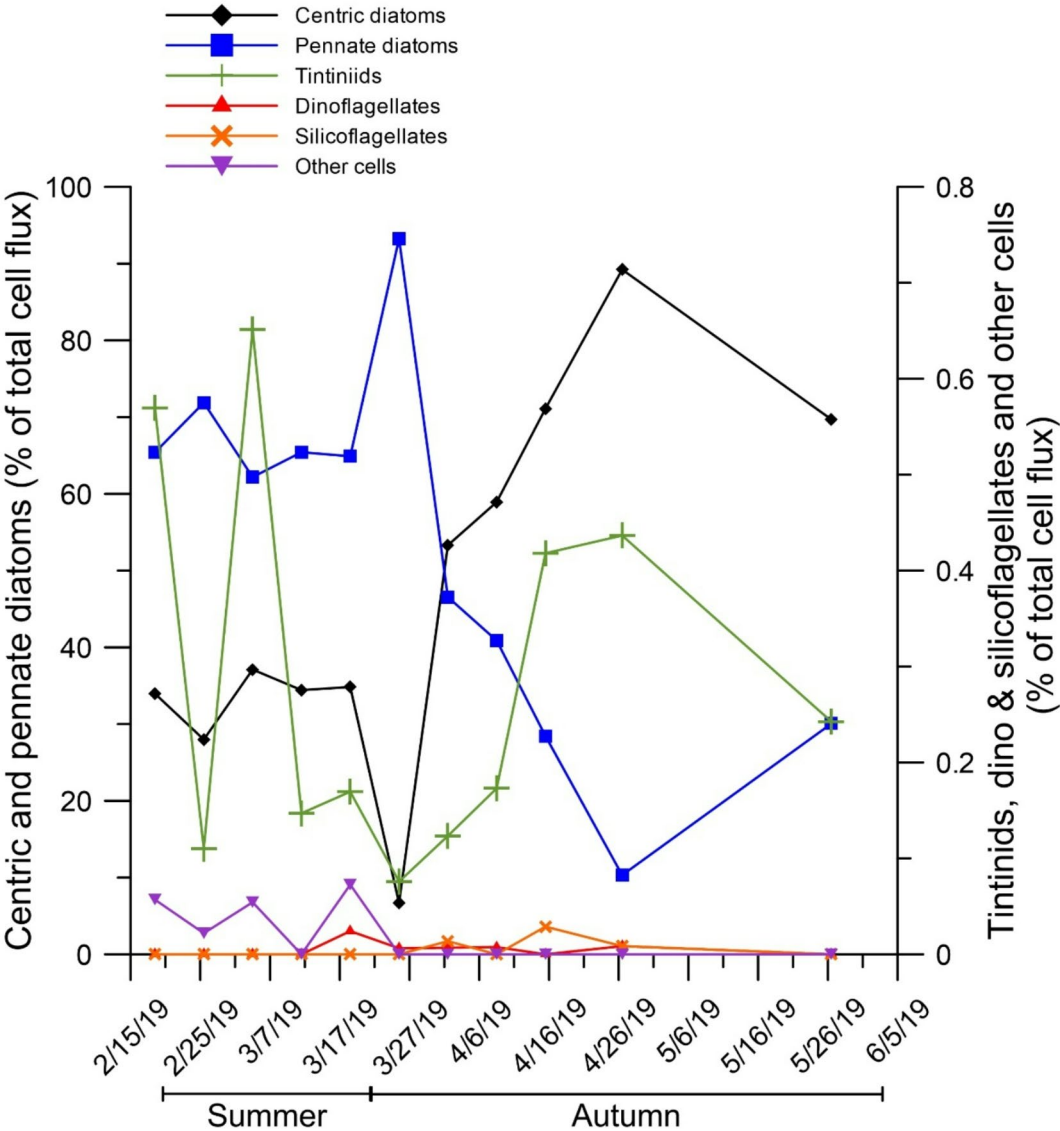
Relative contribution (%) of microplankton functional group percentages to the total microplankton flux. Other cells include foraminifera, radiolarian and phaeodaria.

**Fig. 5:**
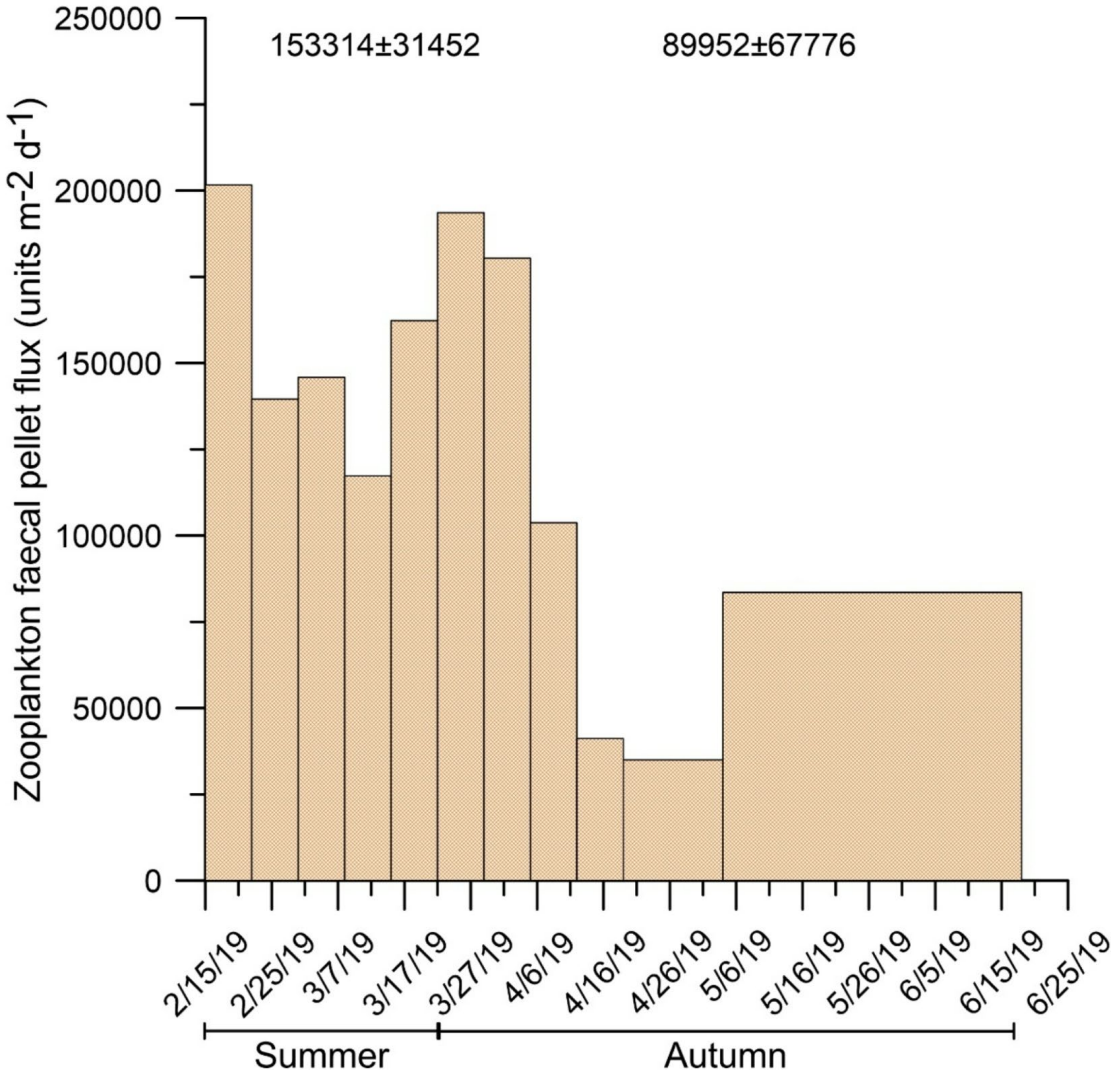
Zooplankton faecal pellet (zFP) flux collected in DISB. The numbers above the histogram indicate the seasonal averages.

**Fig. 6:**
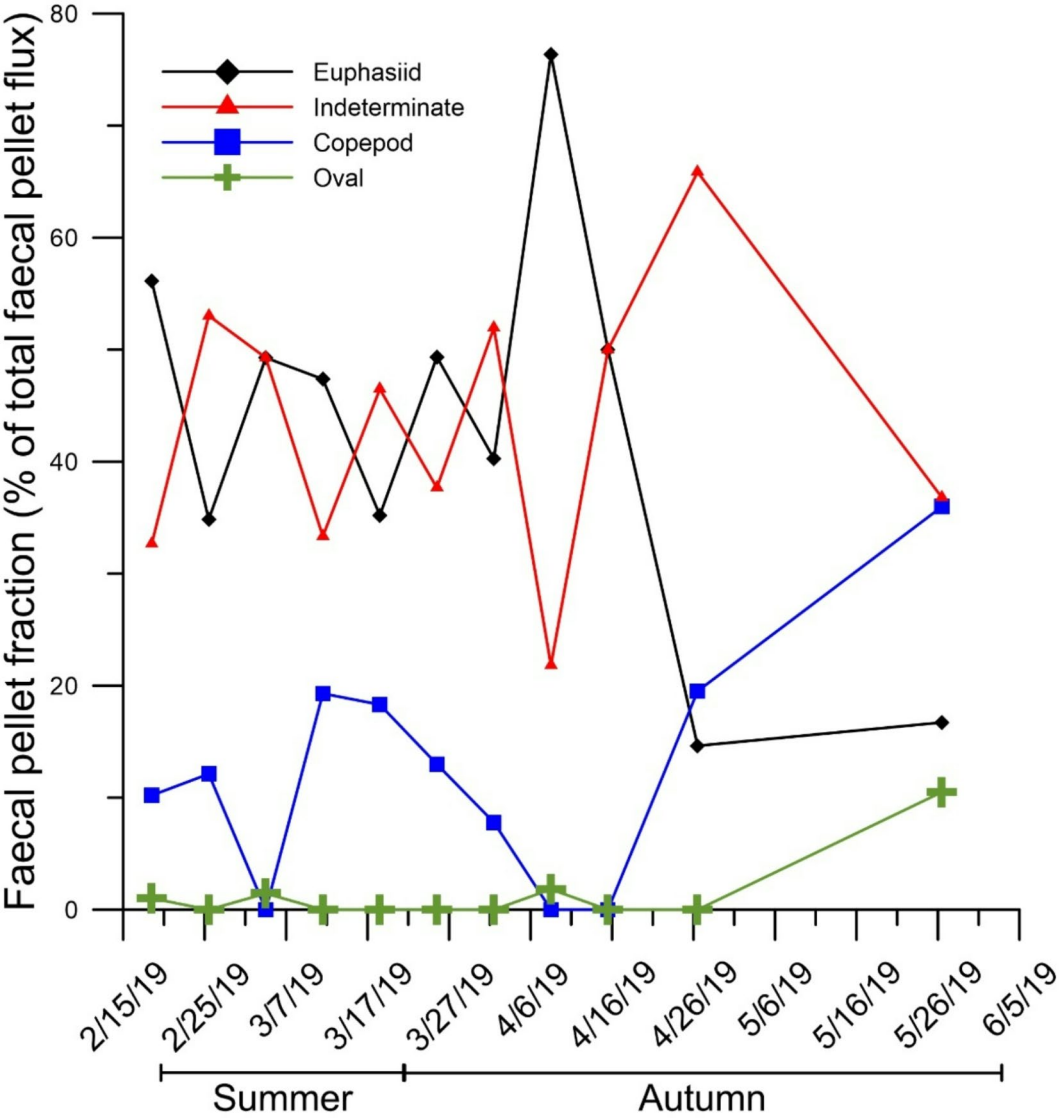
Relative contribution (%) of the different zooplankton faecal pellet groups to the total zooplankton faecal pellet flux.

**Fig. 7:**
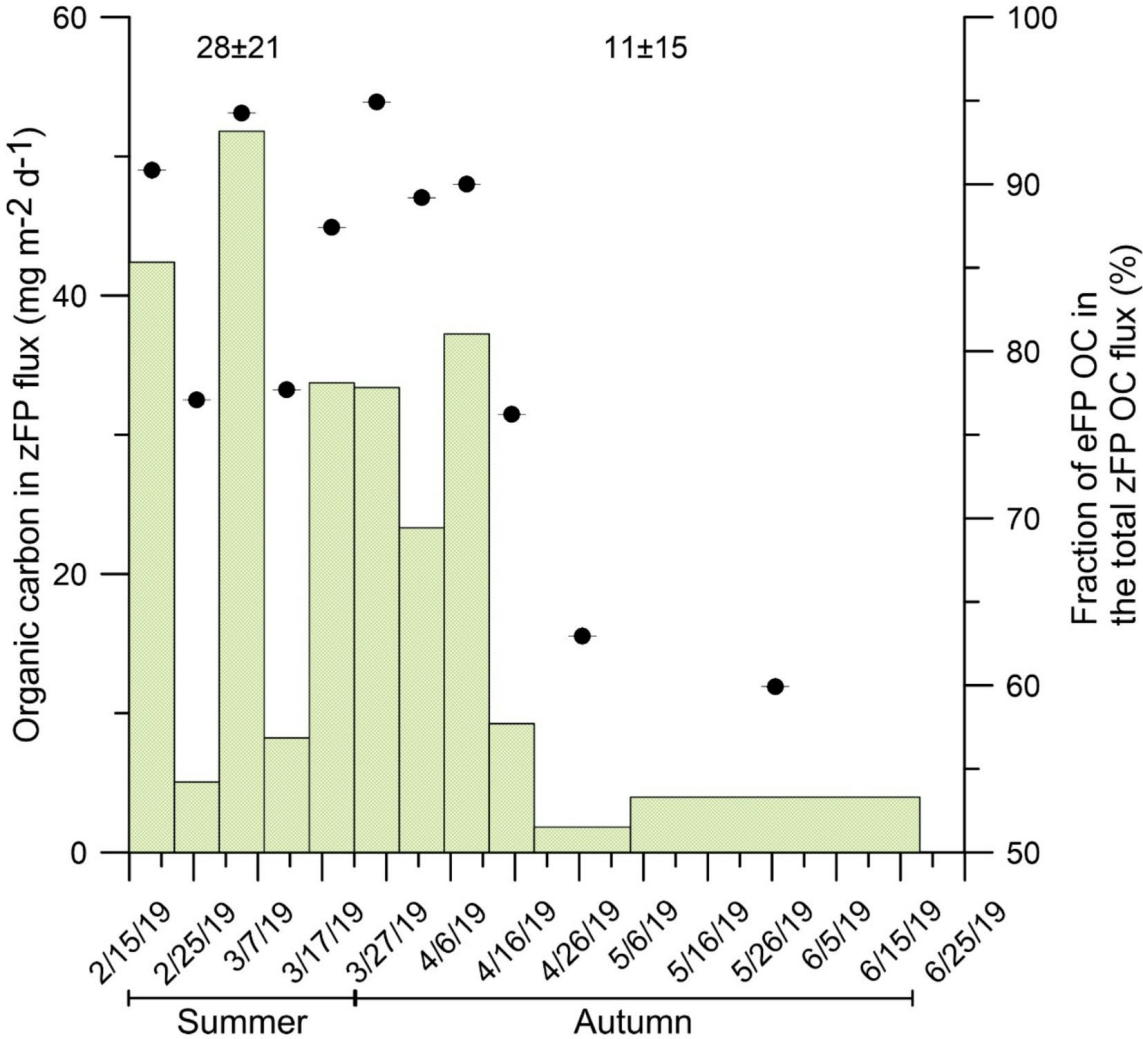
Particulate organic carbon content in the zooplankton faecal pellet (zFP) flux. The numbers above the histogram indicate the seasonal averages. The right X axis and the black dots show the relative contribution (%) of euphausiid faecal pellet (eFP) OC in the total OC zFP flux collected in DISB.

Microplankton functional group fluxes were dominated by pennate diatoms during summer (> 62% of the flux) and centric diatoms during autumn (> 53% of the flux), the rest of the analyzed groups (e.g., dinoflagellates, tintinnids, foraminifera, radiolarian, phaeodaria) represented < 1% of the total microplankton cell flux in the complete study period (Fig. 4 and Supplementary Fig. 3a to f). Centric and pennate diatom abundances along the study showed opposite trends, suggesting a seasonal succession pattern towards the autumn, when *Chaetoceros *spp. (mainly resting spores) and *Thalassiosira *spp. (typically autumn dwelling species)^57,58^ dominated the centric diatom community in DISB (Supplementary Fig. 4). During summer, *Cocconeis *spp. and *Pseudo-nitzschia *spp. were the prominent pennate diatoms, normally succeeding under long-photoperiod and low-salinity conditions^59,60^ (Supplementary Fig. 5). Diatom predominance in the phytoplankton population has been observed in receding sea-ice, wind-sheltered and stratified water column conditions in near-shore areas in the Bransfield and Gerlache Straits^61–64^. These environmental characteristics also occur in DISB to the South of Gerlache Strait^35^, setting conditions for the development of the observed micro-zooplankton cell abundances (Fig. 4). Diatoms have also been associated to ice melting at the eastern coast of the AP and to sub-surface (e.g., 40 to 100 m water depth) chlorophyll-a concentration maxima derived from higher iron concentrations relative to surface values off both coasts of the AP^63,64^. In 2017, chlorophyll-a concentration maxima between 20 and 80 m water depth were also observed for the DISB near the glacier relative to the adjacent oceanic region^35^. Given that the sediment trap in DISB was tethered at 190 m water depth, tens of meters below the observed water depth for the iron-fertilized chlorophyll-a maximum, at least a fraction of the DISB diatom community samples could belong to sub-surface diatom populations. During the summer of 2017, glacier runoffs fertilized DISB with iron and developed shallow freshwater plumes, which strengthen water column stratification and eventually leaded to a diatom bloom^35^. Our results indicated that most likely, these conditions repeated during the present study in the summer of 2019, also implying that summer glacier melting continued during the autumn. Diatom biomass and POC concentration were correlated in coastal WAP waters^65^. In the present study, total diatom flux (e.g., centric and pennate diatom fluxes together) and POC flux were significantly correlated (Figs. 3a and 4 and Supplementary Table 1); however, the centric diatom or pennate diatom fluxes alone did not show a significant correlation with the POC flux. These results showed that the diatom assemblage rather than the dominant functional group (e.g., centric), controlled the phytoplankton POC flux in DISB. Other phytoplankton groups such as tintinnids were present along the complete study period and their flux was significantly higher in summer than in autumn (Supplementary Fig. 3c). Silicoflagellates were only observed during autumn, whereas, dinoflagellates only developed during the transitional weeks between late summer and early autumn, other microzooplankton cells (e.g., foraminifera, radiolarian, phaeodaria) occurred only in the late summer. Recent studies in the Gerlache Strait showed that small flagellates may dominate the abundance in the phytoplanktonic community over the presence of diatoms^19,66^. Together with ongoing ocean warming, one the main factors to explain the observed shift in the phytoplankton community composition in the Gerlache Strait was light availability, where small flagellates seem to successfully grow in highly illuminated waters in shallow upper mixed layers with strong water column stratification conditions. The very low flagellate fluxes (e.g., < 1% of the total microplankton cell flux) observed in the present study, suggest that these conditions did not coincided in DISB. Perhaps, good illumination conditions did not develop due to the shadow of the sediment load from glacier runoffs, in addition to the observed wind mixing^35^. Interestingly, bSi flux was not significantly correlated to any of the diatom fluxes (e.g., centric, pennate or both together); however, it was significantly correlated to the eFP flux, the main constituent of the zFP flux (Figs. 5 and 6, Supplementary Figs. 6a to d, and Supplementary Table 1). This correlation indicates that the intense zooplankton trophic activity, represented by the densely bSi packed (e.g., more concentrated) eFP, is more important than the diatom flux in exporting bSi towards the seabed in DISB. In addition, the diatom content diversity within eFP also showed the effective and indiscriminate euphausiid diatom ingestion and efficient bSi and POC export in Antarctic waters^67^ (Supplementary Fig. 7). Regarding the energy in the total zFP flux, its POC content varied between 12 and 100% of the total DISB POC flux (Figs. 3a and 7). During summer, the POC contained in the zFP flux represented 64% ± 40% of the total seasonal DISB POC flux average, whereas during autumn, the proportion was 38% ± 19%. The total zFP flux, as well as the amount of POC transported in it, showed no significant seasonal differences (Fig. 7, Supplementary Fig. 6 and Supplementary Table 1). However, the total POC flux and the POC in the zFP flux were significantly correlated, in agreement with previous observations in the offshore Bransfield and Gerlache Straits, where eFP were identified as important POC carriers to the seabed^21,26,68,69^. In DISB, the eFP dominated the zFP flux (> 34%; Fig. 6), even more considering that based on their cylindrical shape and texture, the group classified as indeterminate, seemed to correspond to eFP (together > 53% and < 99% of the zFP flux; for indeterminate faecal pellet characteristic details, please see the Material and Methods section). Euphausiid (e.g., krill) dominance in the DISB POC export to the sea floor aligns with observations for open water settings off Anvers Island to the South of Gerlache Strait^26^. In the present study, the POC in eFP represented > 60% (peak of 95% by the end of March) of the POC transported in the total zFP flux, showing the importance of euphausiids (e.g., krill) in the export of OC to the coastal Antarctic seabed (Fig. 7). For the DISB case only, with an area of ~ 2.5 km^2^ (www.add.scar.org; Supplementary Fig. 8) and considering the summer (90 days) and autumn (92 days) temporal extent, together with the zFP and eFP OC flux averages (28 mg m^−2^ d^−1^ and 25 mg m^−2^ d^−1^ and 11 mg m^−2^ d^−1^ and 9 mg m^−2^ d^−1^ for each season, respectively), the amount of POC transported to the seabed via zFP and eFP during both seasons would account for approximately 8.83 ton y^−1^ (3.5 g m^−2^ y^−1^) and 7.7 ton y^−1^ (3.1 g m^−2^ y^−1^), respectively. Using the lowest values (e.g., autumn averages), the spring zFP and eFP productions (36 days, from mid-November to December 21, sensu Gleiber et al.^26^) would add up for 0.99 ton OC y^−1^ (0.4 g OC m^−2^ y^−1^) and 0.81 ton OC y^−1^ (0.3 g OC m^−2^ y^−1^), respectively. Regarding the bSi flux in DISB, the spring to autumn transport towards the seabedwould account for 56 ton y^−1^ (22.4 g m^−2^ y^−1^). The zFP flux autumn average was smaller than in summer (Fig. 5), coincident with a reduction in the proportion of eFP abundance (Fig. 6). Albeit the lack of significant differences among seasons, the zFP flux reduction and the increase in copepod and oval FP abundances during the autumn may reflect eFP degradation due to copepod coprophagy and coprorhexy (fragmentation of pellets)^70–75^. The long, cylindrical krill faecal strings are especially sensitive to fragmentation due to the trophic activity of copepods and other zooplankton groups^74,76^. This eFP fragile condition and the significant relationship among copepod FP and indeterminate zFP abundances (e.g., eFP fragments) suggest that coprophagy and coprorhexy occurs in DISB (Supplementary Table 1). Similarly, the low copepod FP flux and its OC content, (< 7% of the zFP OC flux) observed for DISB, also advocate for copepod coprophagy^77^. The observed zFP sediment trap collections for DISB indicated that krill dominated the macrozooplankton population over salps and copepods. Observations off the WAP to the South of Gerlache Strait, showed that during summer, krill was more abundant than salps and copepods inshore and over the continental shelf, whereas salps predominated offshore at the shelf break^78^. However, to the South of Gerlache Strait, higher copepod abundance relative to krill was observed for Marguerite Bay during the autumn^79^. If this was the case for DISB, the sediment trap collections in the present study only reflect intense copepod coprorhexy/coprophagy albeit, also indicate that eFP are the main zooplankton vehicles transporting OC and bSi to the seabed over copepod FP and oval FP.

### Is climate change at play?

The lack of information on the long term (e.g., decades) annual variation of settling particulate mass and OC fluxes in the Antarctic coastal zone, particularly off the WAP, challenges a robust assessment on the influence of the ongoing climate change on the present results. However, based on the growing evidence on the effects of climate change in the AP vicinity, such as (i) air and sea surface temperature increases^80,81^, (ii) sea ice extent and timing alterations^14,82,83^, (iii) phytoplankton and zooplankton migrations^16,17,84^, (iv) reduction in plankton diversity and carbon drawdown^85^, most likely changes in the temporal variation of settling particulate matter and POC fluxes has also developed. On this line, long-term (e.g., decades) remote sensing observations have shown that during a few decades ago, wind speed has been increasing in contrast to sea ice cover^80,81^, enabling substantial biogenic production beyond the autumn equinox^13^. This seasonal shift demonstrated that phytoplankton phenology is changing at the marginal sea ice in the region, strongly suggesting that the associated flux of settling biogenic particles may experience a concomitant change.

Based on existing records for the shallow Antarctic coastal zone, off Deception Island^34^, it seems that at least since the beginning of the XXI century, the autumnal settling particulate mass and POC fluxes has been similar to their summer counterparts. This coincidence implies that the present observations for Doumer Island represent so far, the southern spatial limit for the vigorous autumnal pelagic production of settling biogenic particles. Due to the ongoing environmental warming trend, glacier runoff most likely will increase and the pelagic POC flux will follow the same tendency. It is projected that warming of WAP open waters will benefit cryptophytes over diatoms; however, the observed shift in the microalgae community composition from diatom to cryptophytes seems not to be taking place in the near-shore DISB yet^65,85^. Indeed, our autumnal observations match with one of the three summer biological production patterns described for the WAP^85^. This is, large centric diatom intensive blooms stimulated by sea-ice melt and a stable iron-rich water column due to glacial discharge fuel a short food chain from krill to top predators^85^. This high-productivity mechanism results in high export production, where only for DISB accounts for an annual budget of > 18 tons of total POC and > 56 tons of bSi towards the seabed. A second production pattern, developing in warmer water, where small cryptophytes dominate over diatoms may result in less efficient POC export towards the benthic environment along a longer food chain, where more trophic level transfers enhance organic matter remineralization^45,77,85^. It seems that this warmer water environment occurs already in offshore waters in the Gerlache Strait^19^. Our results indicated that this scenario is not taking place in DISB yet, highlighting the high spatial variability between Antarctic open waters, channels and semi-enclosed bays. Nevertheless, it should be considered that the routine methodology applied in the present study could have underestimated the flux of the delicate, unarmored cells such as cryptophytes, which could be partially degraded in formaldehyde or not fully counted under 400X microscopy. Overall, the autumnal mass and POC flux observations for DISB matched with summer observations elsewhere in the WAP and the rest of the Antarctic. Assuming that the DISB ecosystem (and potentially the entire coastal Gerlache Strait) moves towards the projected climate warming scenarios, our results suggest that the comparatively high autumn POC flux in DISB relative to Antarctic open water areas could be a previous step to the ongoing displacement of diatoms by cryptophytes observed for WAP open waters, including the Gerlache Strait. Even if the question on whether the current climate change already modified the seasonal pattern of POC (e.g., energy) exports to the seabed will remain open forever, the present study provides basis to show the importance that the autumnal production may have for the maintenance of the open water ecosystem and atmospheric carbon drawdown also beyond the summer. This study may help setting a baseline to assess the ecosystem functioning and carbon budget calculations for the coastal WAP in the XXI century.

## Methods

A mooring line equipped with a Technicap PPS3 sediment trap with a 24-cup rotary carrousel was installed on coordinates 64° 52.325 S, 063° 35.371 W, at the center of Doumer Island’s South Bay (DISB) at 190 m water depth on a station with 240 m water depth and approximately 500 m offshore on a spot directly receiving the island glacier’s melt water inputs (Fig. 1). The original sampling resolution design was set for a complete year; however, due to an equipment failure the sampling period was reduced to 153 days. The sampling period started on February 15, 2019 and ended on June 18, 2019, with sub-sampling periods of 7 to 30 days depending on the season, expecting abundant fluxes in spring and summer and poorer fluxes in autumn and winter (Supplementary Table 2). Sampling cup 11 (4 to 19 May, 2019) was blocked but the material of this sampling period fell into sampling cup 12 (19 May to 18 June, 2019); thus, the collected material in this latter cup corresponds to both sampling periods (45 days).

The Technicap PPS3 sediment trap has an aperture of 0.125 m^2^ and a cylindric-conical shape with an aspect ratio > 4 and a honeycomb baffle system to avoid turbulent outflows at the mouth of the trap and underestimations of mass flux collections. The collecting cups were filled before deployment with a filtered seawater (Whatman GF/F, 47 mm Ø, 0.7 μm pore) buffered solution of 5% formaldehyde and sodium tetraborate. The material from each collecting cup was filter over a 1 mm mesh to remove swimmers (e.g., large zooplankton) that actively got into the trap and subsequently homogenized on a rotary table and split into aliquots with a rotary liquid splitter^86^.

The total mass flux (TMF) was calculated as the product of the total filtered mass weight (mg) times the fraction of the cup sub-sample aliquot divided by the collection area of the sediment trap mouth (0.125 m^2^) times the collection days (7 to 45, depending on the sampling cup setting).

### Microscopical analyses

In the laboratory, aliquots ranging from 0.1 to 1 mL were extracted from each sediment trap cup aliquot using a micropipette after a gentle homogenization and subsequently deposited into a sedimentation chamber (Hydrobios) with a total volume of 3 mL, following the Utermöhl methodology^87^. Subsequently, microplankton cells (e.g., diatoms, dinoflagellates, tintinnids, foraminifera, radiolarian, phaeodaria) were quantified by observing half of the sedimentation chamber under a phase contrast microscope (Zeiss, Axiovert 200) at magnifications of 200X and 400X^88,89^.

Zooplankton faecal pellets (zFP) either intact, broken and preserved fragments, were classified observing the entire sample in the sedimentation chamber. Units were categorized as cylindrical forms (e.g., euphausiids, copepods) and other morphotypes, such as unidentified oval shapes. The group of indeterminate pellets (fragmented or broken fecal strings) could not be definitively classified into any of the previously mentioned groups. However, their morphological characteristics and texture mostly matched those observed for euphausiids FP. Once identified, each FP was measured for length and diameter to determine its volume, using a micrometer ruler placed in one of the eyepieces of the microscope^86,89^.

To analyze the composition of microphytoplankton cells within euphausiid faecal pellets (eFP), 10 to 20 intact eFP (number depending on size, typically 50 to 340 μm in diameter) were removed from the sediment trap samples under a stereomicroscope (Leica model MZ6) at ×4 magnification and placed in a Petri dish with pre-filtered de-carbonated mineral water^86^. After washing, 1 to 10 pellets (number depending on size) were removed from the Petri dish, gently placed in 2.5 mL vials containing 2 mL de-carbonated mineral water and sonicated at 50 to 60 Hz for 30 to 45 s. The contents of the vials were placed in sedimentation chambers where microphytoplankton cells were identified and counted with an inverted microscope (× 1000 magnification). The observed microphytoplankton cells in a known area of the bottom of the chamber were counted and the data extrapolated to the total FP volume.

### Chemical analyses

#### Faecal pellet total and particulate organic carbon

Particulate organic carbon (POC) and ^13^C and ^15^N isotopes were analyzed with an isotope ration mass spectrometer (IRMS) Thermo, Delta V Advantage, coupled to an elemental analyzer Flash EA 2000 in the Biogeochemistry and Applied Stable Isotopes Lab (LABASI) at the Pontificia Universidad Católica (PUC-Chile), using the flash combustion method with acetanilide as standard.

Particulate organic carbon (POC) determination was performed by depositing 0.5 mL to 1 mL the sample onto a Whatmann GF/F glass microfiber filter (~ 0.7 µm pore), subsequently acidified with 2N HCl to remove CaCO_3_. After digestion, excess HCl was eliminated with distilled water and the filter placed in an oven for > 24 h at 50 °C to dry it until reaching constant weight. Faecal pellet POC samples were analyzed in a Europa Hydra 20–20 continuous flow IRMS following combustion at 1000 °C at the UC Davis Stable Isotope Facility Laboratory, using acetanilide as a standard^90^.

The conversion factor to carbon mg eFP used in this study ranged from 0.0088 mg C mm^3^ to 0.021 mg C mm^3^, using an average of 0.017 mg C mm^3^ ± 0.0072 mg C mm^3^. This value was obtained by isolating 180 intact ePF from each sample cup and separating them into 3 GF/F filters, each containing approximately 30 to 80 FP of known volume. Then, each filter was acidified with 2N HCl to remove the CaCO_3_, following the methodology for FP carbon determination^86^. After digestion, the filter was subjected to the same methodology for POC determination described above. The carbon content of copepod FP and oval FP was calculated using a 0.052 mg C mm^3^ transformation factor^72^. The carbon content of indeterminate FP was calculated using a 0.0345 mg C mm^3^ conversion factor, which corresponded to the average of the factors used in this study for copepods and euphausiids. Based on morphological characteristics and texture, we assumed that the indeterminate FP corresponded entirely to fragments of eFP. We also supported this decision on photographic evidence, which showed the overwhelming presence of eFP in the material constituting the total settling mass collected in DISB from a 2-day sediment trap collections at 50 m water depth at the center of the bay (64° 52′ 14.33″ S, 063° 34′ 47,63″ W), during the summer of 2018 (Supplementary Fig. 9). These cylindrical PVC sediment traps have 8 cm in diameter and an aspect-ratio of 12.

Biogenic silica was calculated following molybdate blue spectrophotometric readings (815 nm) after Na_2_CO_3_ sequential alkaline extractions (2 h and 5 h)^30,91,92^ to distinguish the biogenic and lithogenic fractions, which are expressed in weight %.

## Electronic supplementary material

Below is the link to the electronic supplementary material.

Supplementary Material 1

## Data Availability

All data, code, and materials used in the analyses are available in the figures, tables andsupplementary information presented in the article.
