# Supplementary material for: Intense autumnal coastal biogenic particle settling fluxes align with phytoplankton phenology changes off the western Antarctic Peninsula
Source: Sci Rep. 2025 Mar 23;15:10038. doi: 10.1038/s41598-025-92914-9 (PMC11930981; doi:10.1038/s41598-025-92914-9)
Supplement: Supplementary file 1 — Supplementary Material 1 [file 41598_2025_92914_MOESM1_ESM.pdf]

Supplementary Materials for:

Intense autumnal coastal biogenic particle settling fluxes align with phytoplankton phenology changes off the western Antarctic Peninsula

E. Isla, E. Menschel & H. González
*Corresponding author. Email: isla@icm.csic.es

This PDF file includes:

Figures S1 to S9
Tables S1 and S2

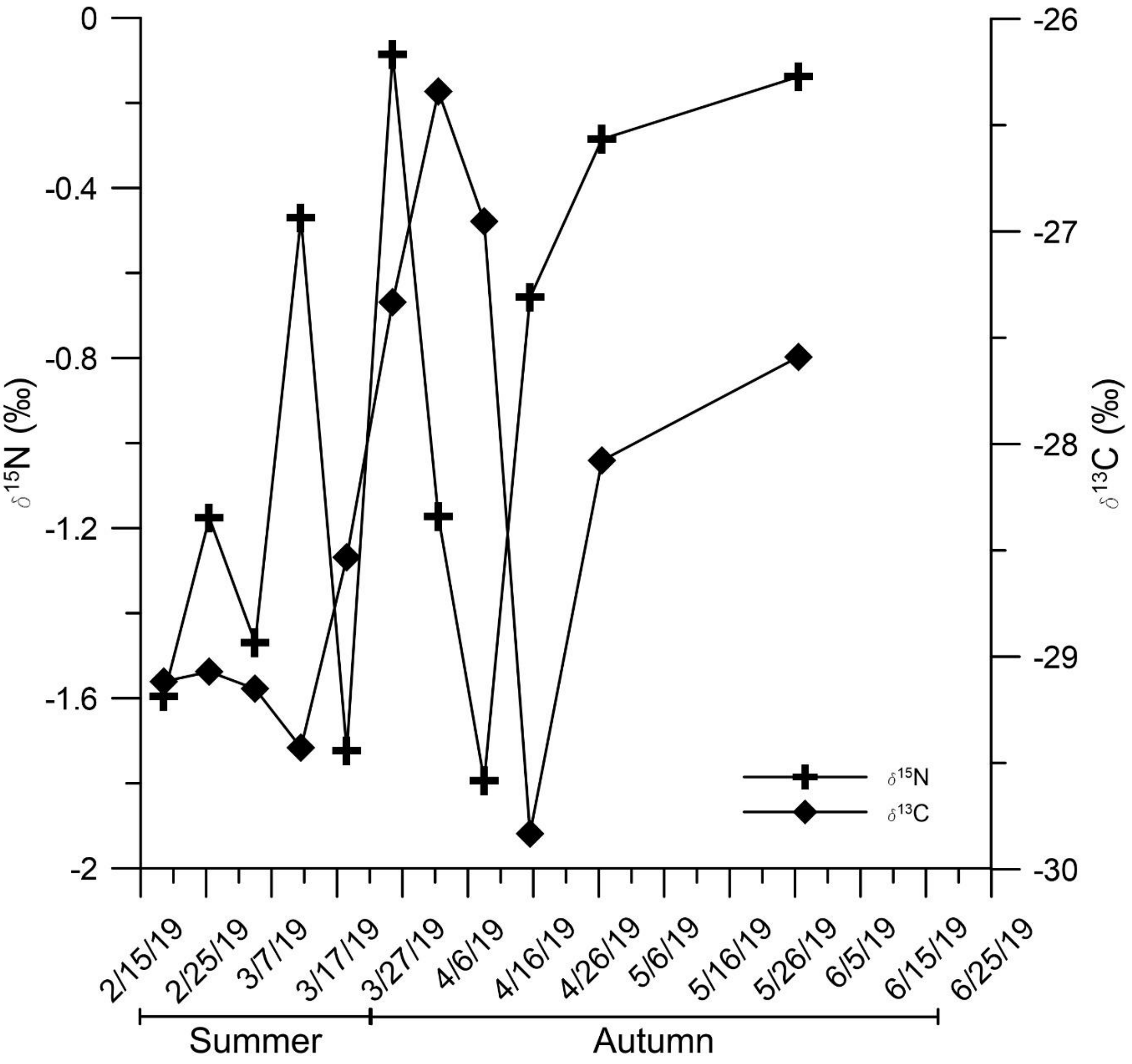

Supplementary figure 1. Carbon (C) and nitrogen (N) stable isotope signals in sediment trap material collected in DISB.

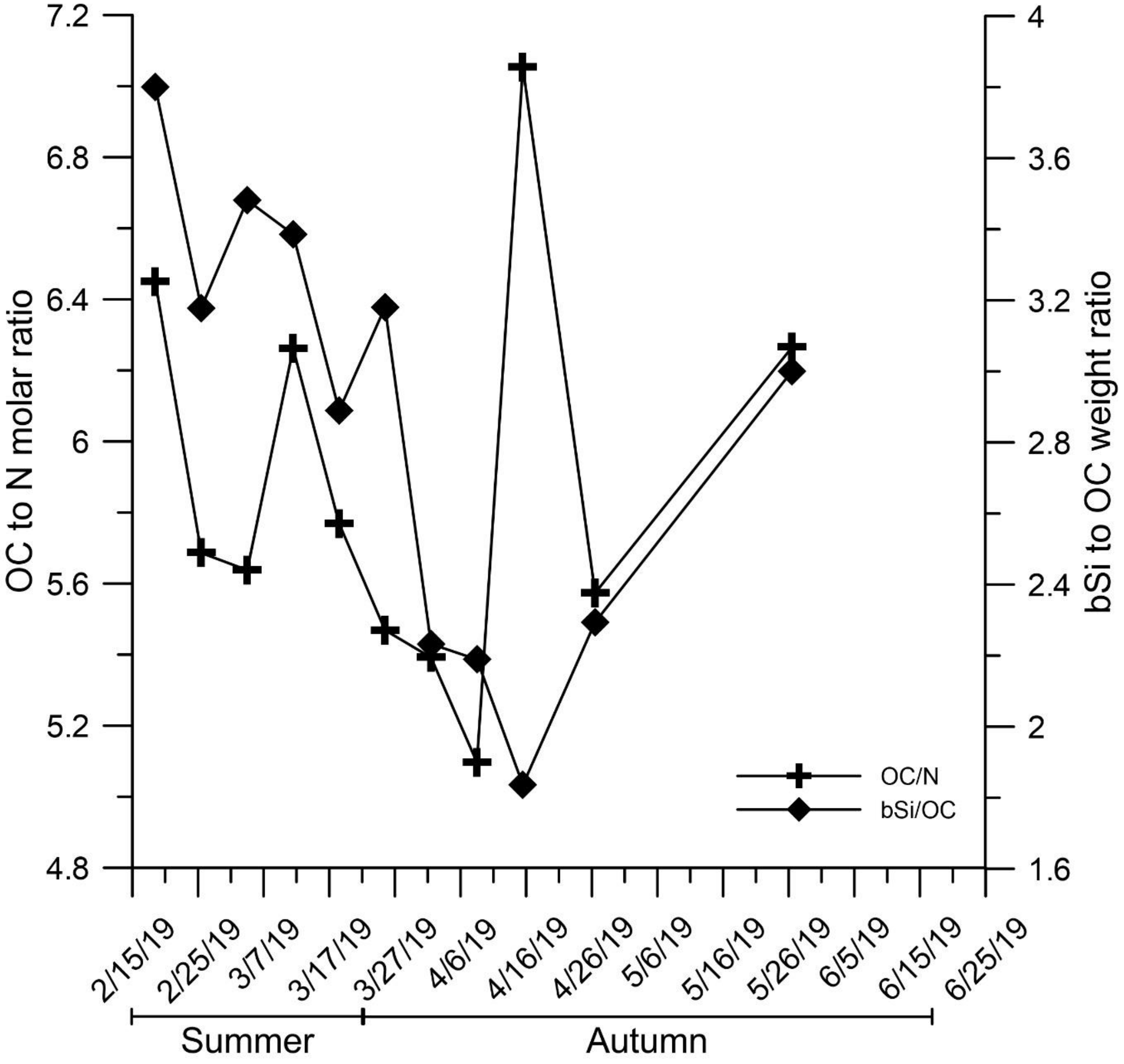

Supplementary figure 2. Organic carbon (OC) to nitrogen (N) molar ratio and biogenic silica (bSi) to OC weight ratio in DISB sediment trap samples.

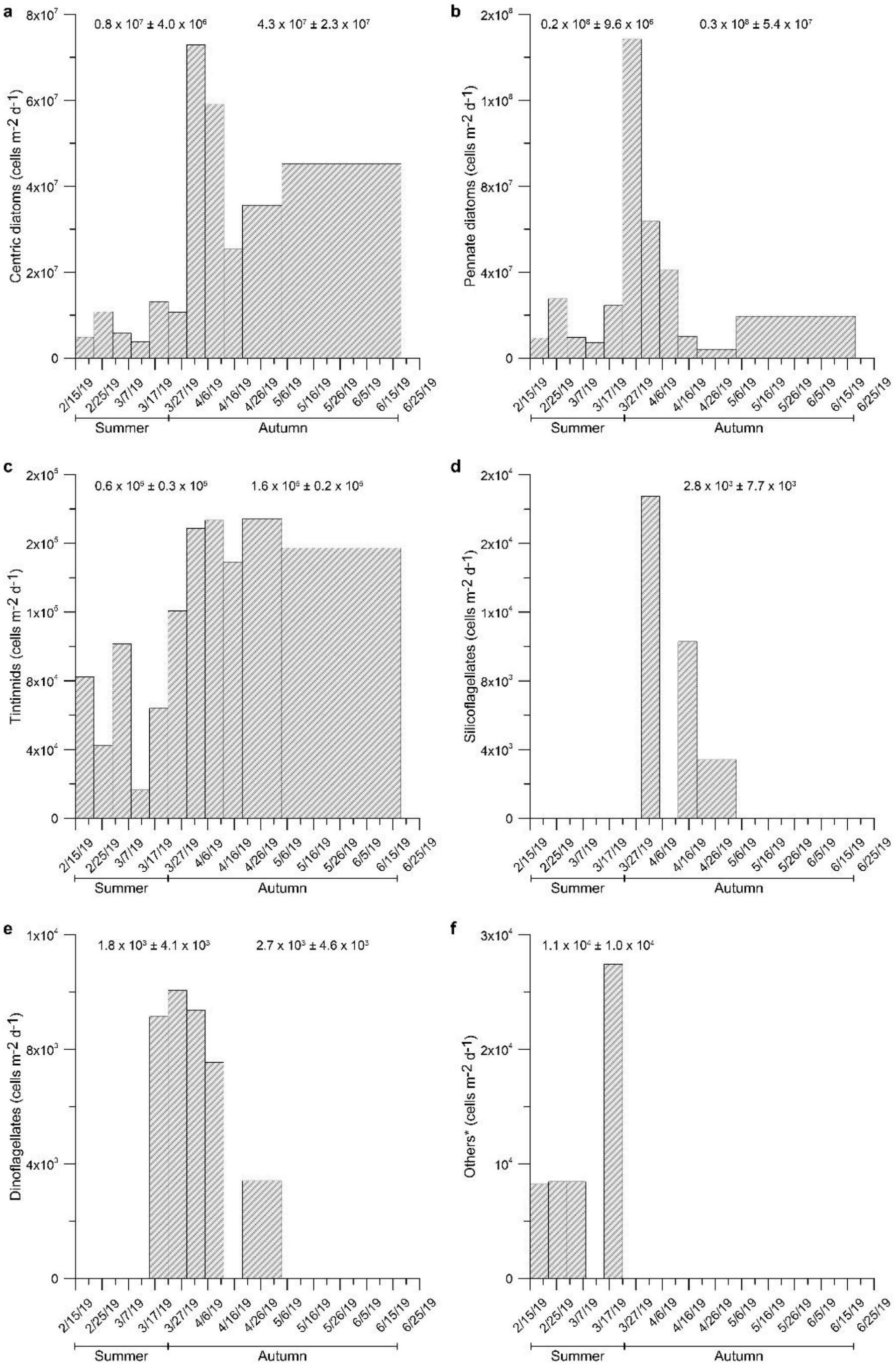

Supplementary figure 3. (a) Centric diatom, (b) pennate diatom, (c) tintinnid, (d) silicoflagellate, (e) dinoflagellate and (f) other cell *(e.g., foraminifera, radiolarian, phaeodaria) fluxes collected in DISB. The numbers above the histogram indicate the seasonal averages.

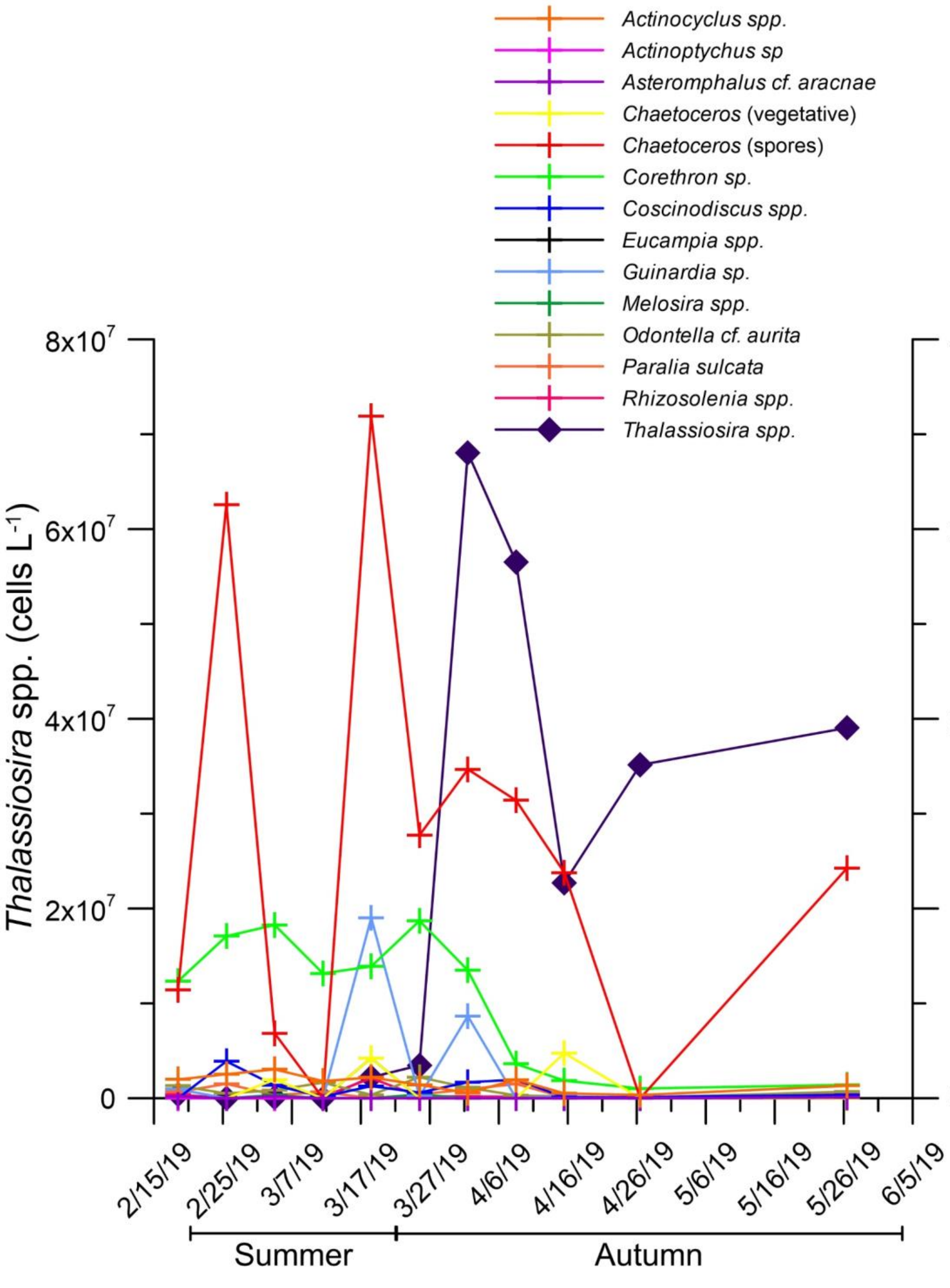

Supplementary figure 4. Main constituents of the centric diatom community collected in DISB. *Thalassiosira spp.* on the X axis has different scale and symbol.

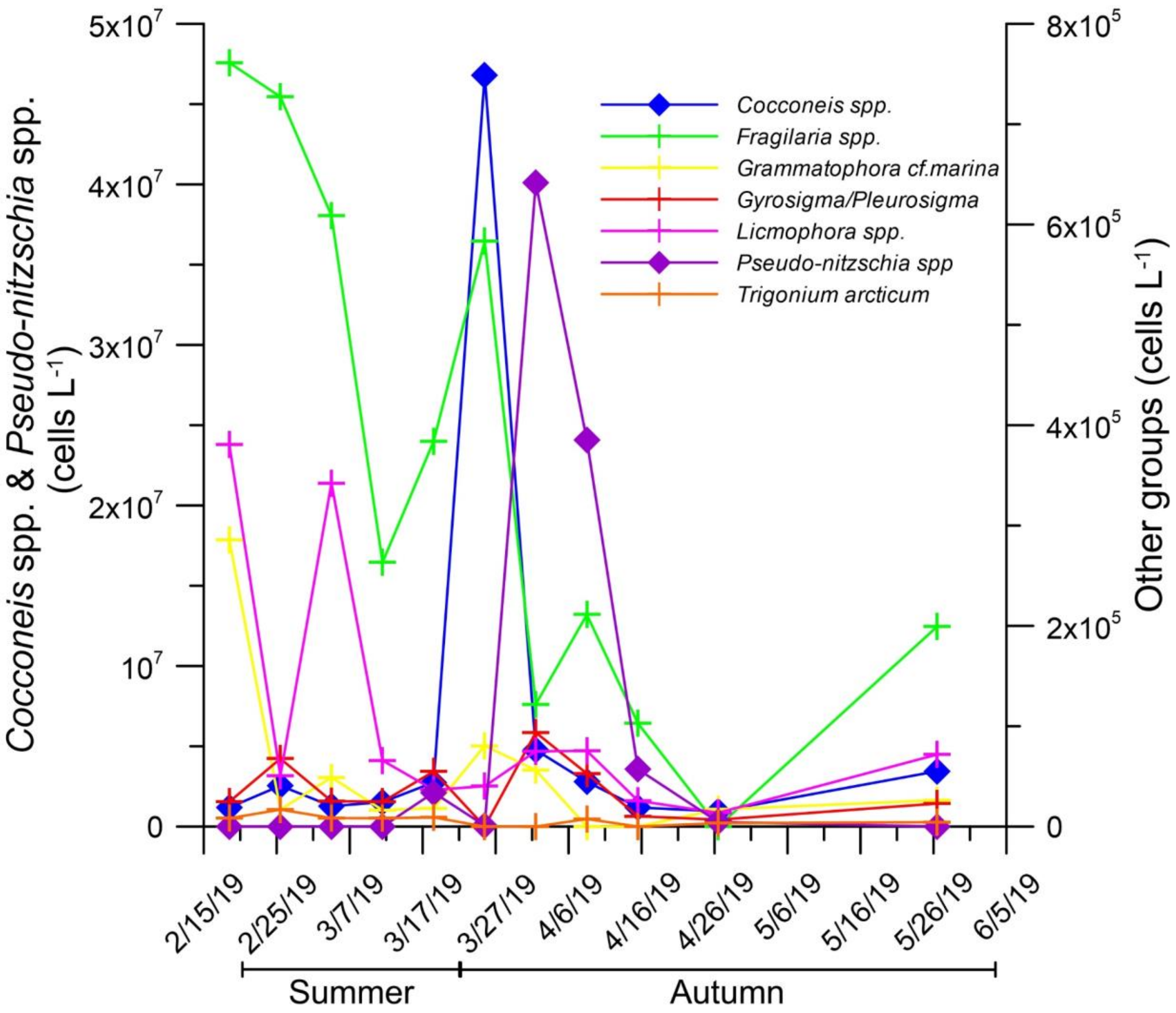

Supplementary figure 5. Main constituents of the pennate diatom community collected in DISB. *Cocconeis spp.* and *Pseudo-nitzschia spp.* on the X axis have different scale and symbol.

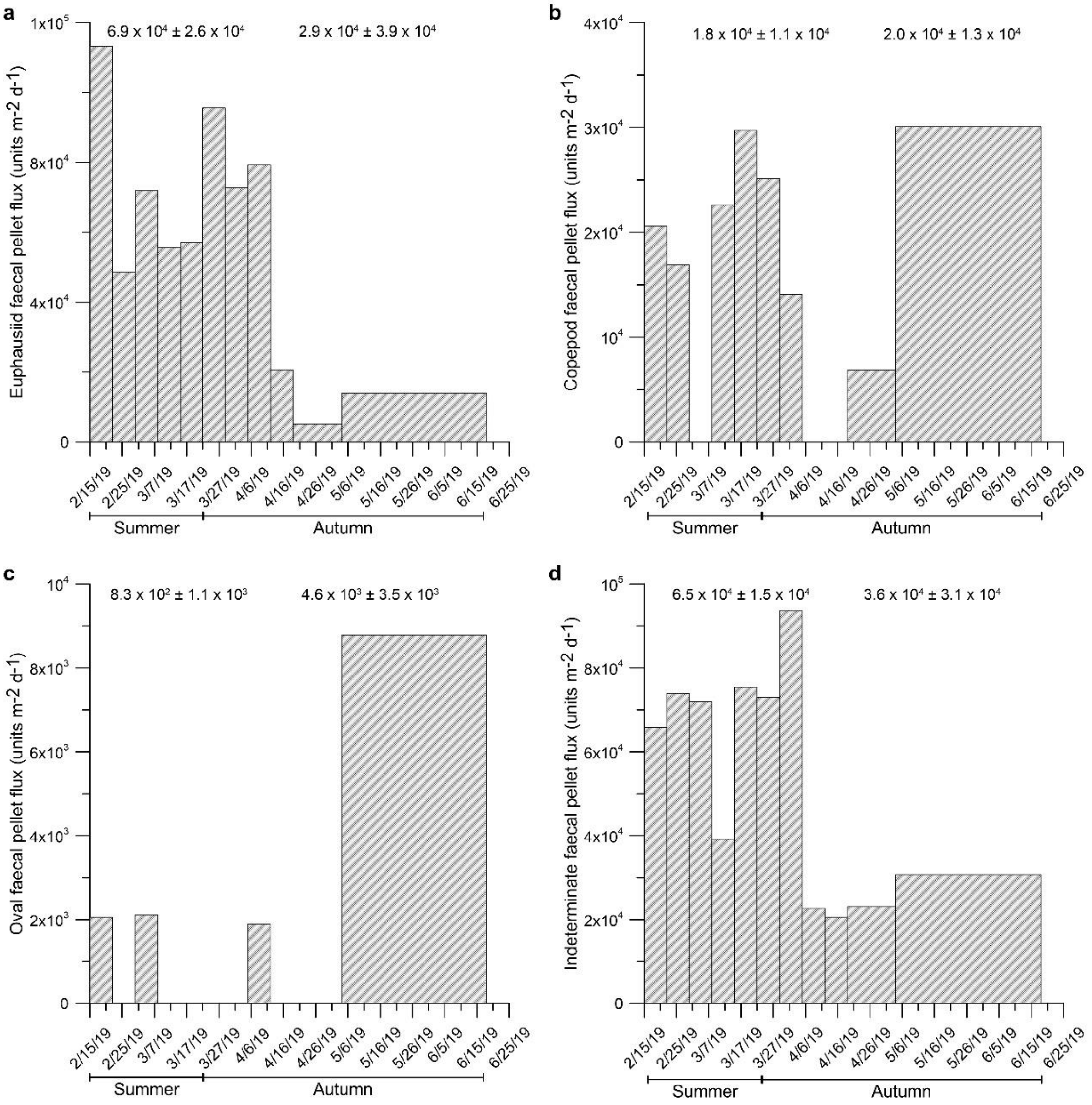

Supplementary figure 6. (a) Euphausiid, (b) copepod, (c) oval and (d) indeterminate faecal pellet fluxes collected in DISB. The numbers above the histogram indicate the seasonal averages.

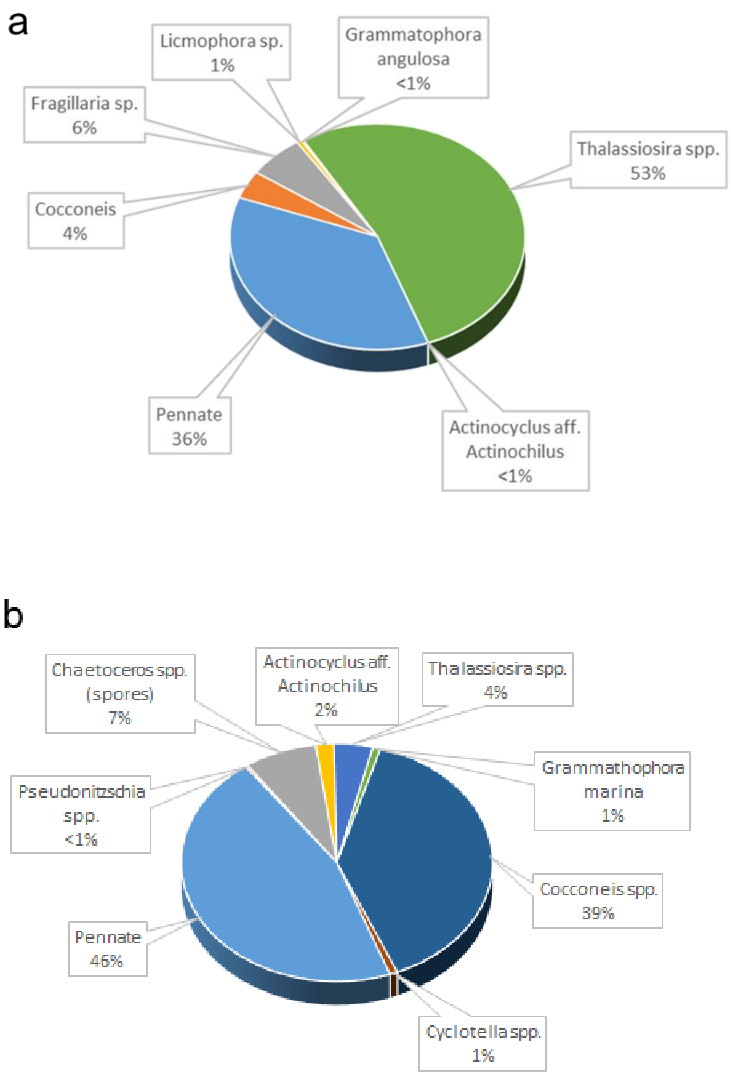

Supplementary figure 7. Microphytoplankton contents in zooplankton faecal pellets collected with sediment trap samples at a) 50 m and b) 200 m water depth in DISB.

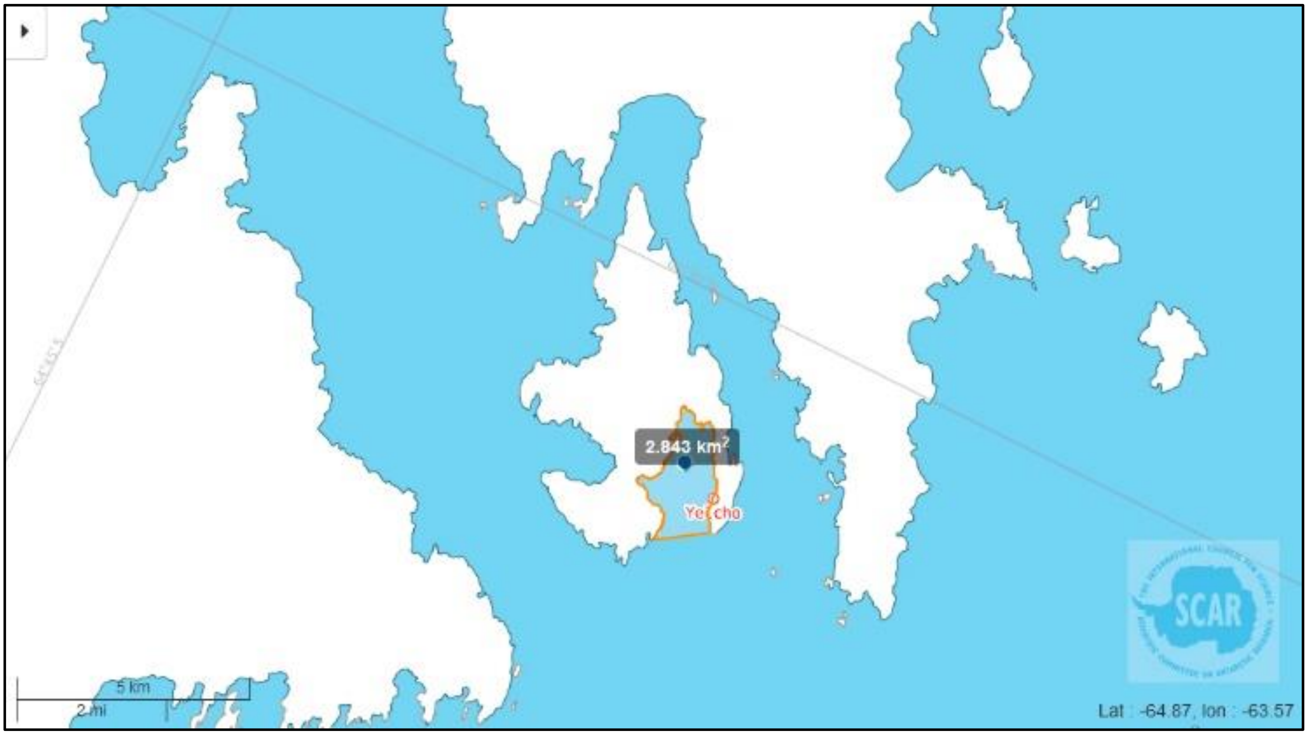

Supplementary figure 8. Doumer Island South Bay area calculated with the Scientific Committee on Antarctic Research's Antarctic Digital Database (2023) Map Viewer software (www.add.scar.org). Coordinates correspond to the blue dot position.

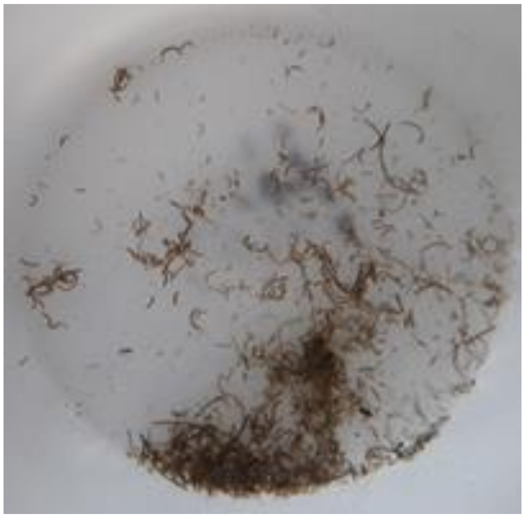

Supplementary figure 9. Settling material collected during 48 h with a sediment trap tethered at 50 m water depth at the center of the Doumer Island’s South Bay (64° 52' 14.33'' S, 063° 34' 47,63'' W), which mostly shows euphausiid faecal pellets.

Supplementary Table S1

Statistical relatioships among variables from sediment trap collections.

All the relationships among variables were tested and only significant relationships are listed here (yellow).

variables	r2	slope	intercept	p value	significant relationship
A vs B	0.85	0.10387	17.364	5.76E-05	yes
A vs D	0.69	0.029868	12.138	0.001616	yes
BvsD	0.7605	0.27864	8.2136	0.000465	yes
BvsO	0.6647	0.24406	-8.9126	0.002227	yes
BvsS	0.6428	0.25269	-7.5382	0.002999	yes
BvsZ	0.7637	888.09	21209	0.000437	yes
BvsAA	0.7849	532.54	-6236.3	0.000283	yes
BvsAD	0.5468	348.15	11902	0.009296	yes
DvsH	0.4332	1.53E+06	-3.06E+07	0.027691	yes
DvsM	0.3901	1.76E+06	-1.36E+07	0.039944	yes
DvsO	0.5276	0.68053	-7.9786	0.011362	yes
DvsS	0.5181	0.71006	-6.7984	0.012503	yes
DvsZ	0.5319	2319.6	31127	0.010867	yes
DvsAA	0.6222	1483.9	-4157.4	0.003907	yes
DvsAD	0.418	952.59	13989	0.031594	yes
D vs AE	0.3904	1.76E+06	-1.37E+07	0.03984	yes
V vs X	0.8176	0.78878	41144	0.000133	yes
AF vs G	0.6152	1.66E+07	4.96E+08	0.004259	yes

A	Total Mass flux (mg m-2 d-1)	R	Undetermined (mg C m-2 d-1)
B	Biogenic silica flux (mg m-2 d-1)	S	Total faecal pellet C flux (mg C m-2 d-1)
C	Biogenic silica content (%)	T	Fecal pellet (units)
D	Organic C flux (mg m-2 d-1)	U	Euphausiid (units)
E	Organic C content (%)	V	Copepod (units)
F	Microplankton flux (cells m-2 d-1)	W	Oval (units)
G	Centric diatoms (cells m-2 d-1)	X	Undetermined (units)
H	Pennate diatoms (cells m-2 d-1)	Y	Total
I	Tintiniids (cells m-2 d-1)	Z	Fecal pellet flux (units m-2 d-1)
J	Dinoflagellate (cells m-2 d-1)	AA	Euphausiid (units m-2 d-1)
K	Silicoflagellate (cells m-2 d-1)	AB	Copepod (units m-2 d-1)
L	Others (cells m-2 d-1)	AC	Oval (units m-2 d-1)
M	Total microplankton flux (cells m-2 d-1)	AD	Undetermined (units m-2 d-1)
N	Fecal pellet (mg C m-2 d-1)	AE	centric + pennate cell flux (cells m-2 d-1)
O	Euphausiid (mg C m-2 d-1)	AF	13C ‰
P	Copepod (mg C m-2 d-1)	AG	15N ‰
Q	Oval (mg C m-2 d-1)		

Supplementary Table 2. Sampling schedule for the sediment trap anchored in the South Bay, Doumer Island, Antarctica.

Sample number	Sampling days	Start Date
1	7	15 feb 2019
2	7	22 feb 2019
3	7	1 mar 2019
4	7	8 mar 2019
5	7	15 mar 2019
6	7	22 mar 2019
7	7	29 mar 2019
8	7	5 Apr 2019
9	7	12 Apr 2019
10	15	19 Apr 2019
11	15	4 may 2019
12	30	19 may 2019
-	-	18 jun 2019 (end date)
